# Glucocorticoids reduce renal NHE8 expression

**DOI:** 10.1002/phy2.31

**Published:** 2013-07-15

**Authors:** Catherine Joseph, Jyothsna Gattineni, Vangipuram Dwarakanath, Michel Baum

**Affiliations:** 1 Department of Pediatrics, University of Texas Southwestern Medical Center at DallasDallas, Texas, 75235-9063; 2 Department of Internal Medicine, University of Texas Southwestern Medical Center at DallasDallas, Texas, 75235-9063

**Keywords:** Na/H exchange, postnatal renal development, proximal tubule, renal acidification

## Abstract

The proximal tubule reabsorbs most of the filtered bicarbonate which is mediated in large part by Na^+^/H^+^ exchange (NHE). We have previously demonstrated that there is an isoform switch during postnatal maturation from NHE8 to NHE3 that is concordant with the postnatal increase in serum glucocorticoid levels. To examine if glucocorticoids may be responsible for this isoform switch, we administered dexamethasone daily to mice at 7–10 days of age, a time prior to the normal isoform switch. We show that compared to vehicle-treated controls, dexamethasone caused a premature increase in renal NHE3 and decrease in NHE8 mRNA, total protein, and brush border membrane protein abundance. To examine if there was a direct epithelial action of dexamethasone on NHE8, we studied normal rat kidney (NRK) cells in vitro which express NHE8 on their apical membrane. Dexamethasone decreased NHE8 mRNA, total protein, and apical protein abundance. Dexamethasone also decreased Na^+^/H^+^ exchanger activity. These studies provide evidence that glucocorticoids may play a role in the developmental isoform switch from NHE8 to NHE3 and cause a decrease in NHE8 expression and activity.

## Introduction

The proximal tubule reabsorbs 80% of the filtered bicarbonate. Luminal proton secretion for bicarbonate reclamation is mediated predominantly by Na^+^/H^+^ exchange (NHE) and to a lesser extent by a H^+^-ATPase (Rector 1983; Preisig et al. 1987). In the adult proximal tubule luminal Na^+^/H^+^ exchanger activity is mediated by NHE3 (Wu et al. 1996; Schultheis et al. 1998; Wang et al. 1999). The adult proximal tubule also expresses another apical Na^+^/H^+^ exchanger designated NHE8 that likely does not play a significant role in the adult proximal tubule acidification (Wu et al. 1996; Goyal et al. 2005).

The rate of bicarbonate absorption in the neonate is less than in adults (Schwartz and Evan 1983; Baum and Quigley 1993b), although virtually all of luminal proton secretion is mediated by a Na^+^/H^+^ exchanger and not by the apical H^+^-ATPase (Baum 1992). The increase in both bicarbonate absorption and Na^+^/H^+^ exchanger activity parallel to each other and adult levels are reached at the time of weaning (Schwartz and Evan 1983; Baum 1990). While the rate of apical membrane Na^+^/H^+^ exchanger activity in neonates is one fourth that of adults (Baum 1990; Shah et al. 2000), NHE3 mRNA and protein abundance is barely detectable (Shah et al. 2000; Becker et al. 2007; Twombley et al. 2010). The disparity between Na^+^/H^+^ exchanger activity and NHE3 abundance is likely due to the presence of NHE8 which is highly expressed on the apical membrane of the proximal tubule of neonates (Becker et al. 2007; Twombley et al. 2010).

We have previously examined the ontogeny of renal NHE3 and NHE8 mRNA and proximal tubule brush border membrane protein expression during postnatal renal development (Becker et al. 2007; Twombley et al. 2010). There is a developmental isoform switch from NHE8 to NHE3 that occurs at about the time of weaning, which is concordant with a 25-fold increase in serum glucocorticoid levels (Henning 1978). We have shown previously that neonatal NHE3 mRNA and protein expression increases prematurely with the administration of glucocorticoids to neonates (Baum et al. 1995; Gupta et al. 2001). The purpose of the present study was to determine whether glucocorticoids are a factor in the maturational decrease in NHE8 and if glucocorticoids affect NHE8 activity.

## Methods

### Animal studies

Animal studies were conducted using neonatal C5B7/BL6 mice. All animal experiments were conducted with approval from Institutional Animal Care and Use Committee at University of Texas Southwestern Medical Center and in keeping with American Physiological Society's Guiding Principles in the Care and Use of Animals. Dexamethasone (6 μg/100 g body weight) or vehicle was injected subcutaneously once daily in neonatal mice starting on day 7 of life for three consecutive days. At 10 days of age, mice were sacrificed and the kidneys were harvested quickly. The harvested tissue was used for preparation of mRNA, total protein, and brush border membrane vesicles (BBMV).

### Total protein and BBMV isolation

Neonatal kidneys were placed in ice-cold PBS and the renal capsule was removed. Approximately 25 mg of tissue set aside for RNA isolation and the remaining tissue placed into isolation buffer (300 mmol/L mannitol, 16 mmol/L 4-(2-hydroxyethyl)-1-piperazineethanesulfonic acid [HEPES], and 5 mmol/L ethyleneglycol-bis(β-aminoethyl)-N,N,N',N'-tetraacetic acid that was titrated to a pH of 7.4 with Tris) with phenylmethylsulfonyl fluoride 10 μg/mL and 1 μg/mL protease inhibitor cocktail (Sigma, St. Louis, MO). The kidney was homogenized with a Teflon-glass homogenizer, at 4°C with 15 strokes. The homogenate (200 μL) was aliquoted for assessment of NHE3 and NHE8 in total protein abundance. The remaining mixture was used to prepare BBMV.

For BBMV isolation, the homogenate was precipitated twice using 1 mol/L MgCl_2_, as previously described (Twombley et al. 2010). The supernatant was collected and MgCl_2_ precipitation was repeated once again. Then the final supernatant was collected into polycarbonate tubes for centrifugation at 20,000 rpm at 4°C for 30 min. This yielded a pellet which was then resuspended in adequate volume of isolation buffer. The protein content of the brush border vesicle thereby collected was estimated by Bradford assay with albumin as a standard.

### Cell culture

Normal rat kidney cells (NRK cells) were purchased from American Type Culture Collection (Manassas, VA). Cells were cultured in high-glucose Dulbecco's modified eagle medium (DMEM) (Gibco, Grand Island, NY) supplemented with 5% fetal calf serum and with 1 mmol/L sodium pyruvate, penicillin (100 U/mL) and streptomycin (100 U/mL) at 37°C in a 95% O_2_/5% CO_2_ environment (Zhang et al. 2007; Joseph et al. 2012). The cells were rendered quiescent when confluent by incubation in serum-free DMEM/Ham's F12 (Sigma, St. Louis, MO). Dexamethasone (10^−6^ mol/L) or vehicle was added 24 h prior to experiments. In experiments designed to examine if NHE8 RNA synthesis was affected by dexamethasone, actinomycin D (5 × 10^−6^ mol/L) was added 3 h prior to the addition of dexamethasone or vehicle.

### SDS-PAGE immunoblotting

The protein obtained from brush border vesicle or total lysates were mixed with 5× loading buffer (2.5 mM Tris HCl [pH 6.8], 2.5% β-mercaptoethanol, 25% glycerol, and 2.5% SDS). The proteins were heated to 85°C for 5 min (NHE3) and 37°C for 5 min (NHE8) and then loaded on an 8% polyacrylamide gel and separated using SDS-PAGE (sodium dodecyl sulfate polyacrylamide gel electrophoresis) as has been described (Baum et al. 2012; Joseph et al. 2012). Proteins were then transferred to a polyvinylidene difluoride membrane (Immobilon; Millipore, Billerica, MA) at 400 mA for 1 h at 4°C (Baum et al. 1998; Shah et al. 2000; Bobulescu et al. 2005). The blots were blocked with Blotto (5% nonfat milk in phosphate buffered saline [PBS], pH 7.4) for at least 1 h before incubation with primary antibodies to either NHE3 or NHE8. The NHE8 antibody was a monoclonal antibody 7A11 at a 1:5 dilution (Pierce, Rockford, IL) and the NHE3 antibody, 3H3 at 1:5 dilution (a gift of Dr. Orson Moe). The blots were washed in 0.05% Tween 20 in PBS followed by the addition of the secondary antibody, horseradish peroxidase-conjugated donkey anti-mouse antibody at a 1:10,000 dilution. Enhanced chemiluminescence was used to detect bound antibody (Amersham Biosciences, Piscataway, NJ). Equal loading of the samples was verified using an antibody to β-actin at 1:15,000 dilution for total lysates (Sigma Biochemicals and Reagents, St. Louis, MO). Villin at 1:250 dilution, an actin-binding protein expressed on brush borders, was used to verify equal loading for brush border vesicles (Santa Cruz Biotech, CA). Relative NHE3, NHE8 to Villin, or β-actin protein abundance were quantitated using densitometry.

For NRK cells were rinsed with PBS followed by radio-immunoprecipitation assay (RIPA) lysis buffer (150 mmol/L NaCl, 50 mmol/L Tris^.^HCl [pH 7.4], 5 mmol/L ethylenediaminetetraacetic acid, 1% SDS, 1% TritonX-100, and 0.5% deoxycholate containing protease inhibitors). The lysates were centrifuged at 14,000*g* for 30 min at 4°C and the protein content was measured using the Bradford reaction and processed as above.

### NRK NHE8 surface expression

Cell surface biotinylation has previously been described by our laboratory (Bobulescu et al. 2005; Joseph et al. 2012). NRK cells were rinsed with ice-cold PBS and then biotinylated with 1.5 mg/mL sulfo-NHS-SS-biotin (Pierce, Rockford, IL) in 10 mmol/L triethanolamine (pH 7.4), 150 mmol/L NaCl, and 2 mmol/L CaCl_2_. The cells were then incubated at 4°C for 60 min on a horizontal rotator. The cells were subsequently washed twice with quenching buffer (PBS with 100 mmol/L glycine, 1 mmol/L MgCl_2_, and 0.1 mmol/L CaCl_2_) for 20 min at 4°C followed by a final wash with PBS. The cells were lysed with RIPA buffer containing protease inhibitors and centrifuged at 13,000*g*. The supernatant was diluted to 2.5 mg/ml protein with RIPA buffer containing protease inhibitors. Equal amounts of protein lysates were then incubated at 4°C overnight with streptavidin-agarose beads (Pierce, Rockford, IL). The beads were washed and biotinylated proteins were heated to 85°C in loading buffer which released the proteins from the beads (Bobulescu et al. 2005; Zhang et al. 2007). Proteins were separated using SDS-PAGE and processed as above.

### cDNA synthesis and real-time PCR

Total cellular RNA was isolated from NRK cells or renal cortex (25 mg) using GenElute Mammalian Total RNA Miniprep Kit as per the manufacturer's instructions (Sigma-Aldrich, St. Louis, MO). The RNA quality and concentration were assayed using a microplate spectrophotometer (Biotek,Winooski, VT). RNA (2 μg) was treated with DNAse I and was used to synthesize cDNA using random hexamer primers and reverse transcriptase (Stratagene, La Jolla, CA) at an annealing temperature of 25°C for 10 min, extension at 42°C for 52 min, and termination at 70°C for 15 min.

mRNA abundance was assessed by real-time polymerase chain reaction (PCR) using an iCycler PCR Thermal Cycler (BioRad, Hercules, CA). Primers for NHE8 were mixed with cDNA and SYBR green master mix (BioRad) as per the manufacturer's instructions. The PCR conditions were denaturation at 94°C for 30 sec, annealing at 61°C for 20 sec, and extension at 72°C for 20 sec for 40 cycles. Housekeeping gene 28s was used to normalize relative expression of NHE3 and 8 (Vandesompele et al. 2002). For in vivo studies using NHE3, we used forward primer 5′-TTCAAATGGCACCACGTCCAGG-3′ and reverse primer 5′-TGACCTTGTGGGACAGGTGAAAG-3′ and for NHE8 5′-ACAGTTTCGCATTTGGCTCCCTG-3′ as forward primer and 5′-TGTTGGTGAGGACGATGGAGACTG-3′ as reverse primer. NRK cell NHE8 primers were (forward) 5′-AAGCCTATTCTTCCGGTGCAGACA-3′ and (reverse) 5′-AGAGAAACAACAGCCACGCTCTCA-3′. The forward primer for 28s was 5′-TTGAAAATCCG-GGGGAGAG-3′ and reverse primer was 5′-ACATTGTTC-CATGCCAG-3′ for mouse and NRK studies.

### Na^+^/H^+^ exchanger activity

The pH-sensitive dye 2′,7′-bis(2-carboxyethyl)-5(6)-carboxy fluorescein (BCECF-AM, Molecular Probes, Eugene, OR) was used to measure NHE8 activity (Zhang et al. 2007; Gattineni et al. 2008; Joseph et al. 2012). Confluent NRK cells were grown on coverslips and then incubated with 10 μmol/L BCECF-AM (BCECF-acetoxymethyl ester) for 20 min at 37°C. Intracellular pH (pHi) was determined from the ratio of fluorescence (excitation: 500 and 450 nm, emission: 530 nm) in a computer-controlled spectrofluorometer (Photon Technology International, Birmingham NJ). The fluorescence ratio was assessed using the K^+^/nigericin technique at 500/450 to measure pHi (Moe et al. 1991; Baum et al. 1993).

To measure NHE activity, NRK cells were initially incubated in sodium-free solution with nigericin (10 μg/mL) in the absence of CO_2_/HCO_3_^−^ resulting in cell acidification due to the absence of sodium and the cell-to-bath potassium gradient. The sodium-free solution consisted of (in mmol/L) 115 choline chloride, 5 KCl, 1.54 MgCl_2_, 1.1 CaCl_2_, and 30 HEPES. This and all solutions had pH 7.40 and osmolality 295 mOsm. After a steady state pH was reached, the nigericin was removed by replacing the solution with a sodium-free solution containing albumin (1 g/dL). There was no change in cell pH until the sodium-free solution was replaced by a sodium-containing solution (in mmol/L) 25 NaCl, 90 choline chloride 5 KCl, 1.54 MgCl_2_, 1.1 CaCl_2_, and 30 HEPES. The initial rate of change in pHi upon addition of sodium was due to NHE and the rates under various conditions were compared (Zhang et al. 2007; Gattineni et al. 2008; Joseph et al. 2012).

### Statistical analysis

All data are expressed as mean ± SEM. Comparisons were made using an unpaired Student's *t*-test. A *P* value of ≤0.05 was considered significant.

## Results

**Figure 1:**
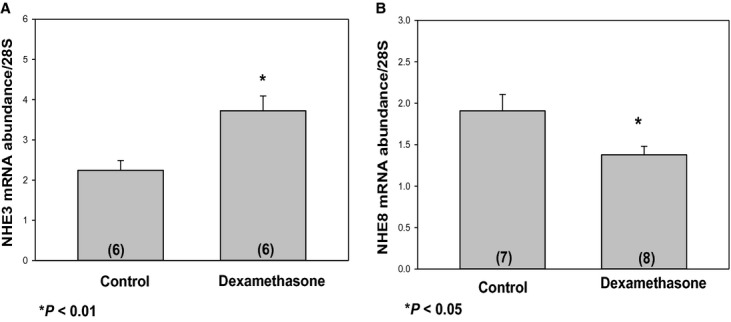
Effect of dexamethasone on neonatal renal NHE3 and NHE8 mRNA abundance. Neonatal mice were administered dexamethasone (6 μg/100 g body weight) or vehicle subcutaneously daily for 3 days starting on day 7 of age and kidneys were harvested on day 10. mRNA abundance was assessed using real-time PCR. Administration of dexamethasone resulted in an increase in NHE3 mRNA abundance (A) and decrease in NHE8 mRNA abundance (B).

**Figure 2:**
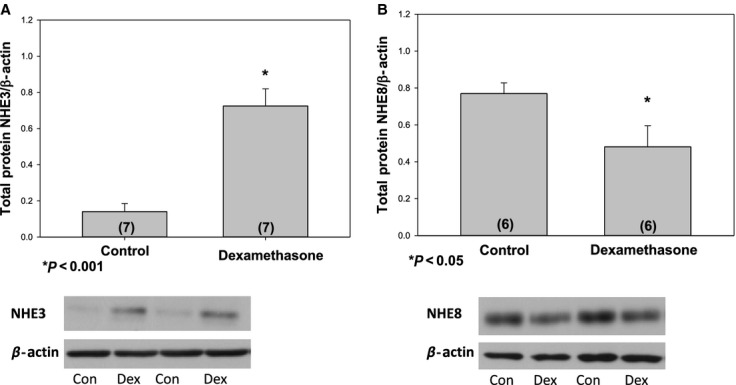
Effect of dexamethasone on neonatal renal NHE3 and NHE8 total protein abundance. Neonatal mice were administered either vehicle or dexamethasone. Neonatal administration of dexamethasone resulted in an increase in NHE3 (A) and decrease in total kidney NHE8 (B) total protein abundance.

**Figure 3:**
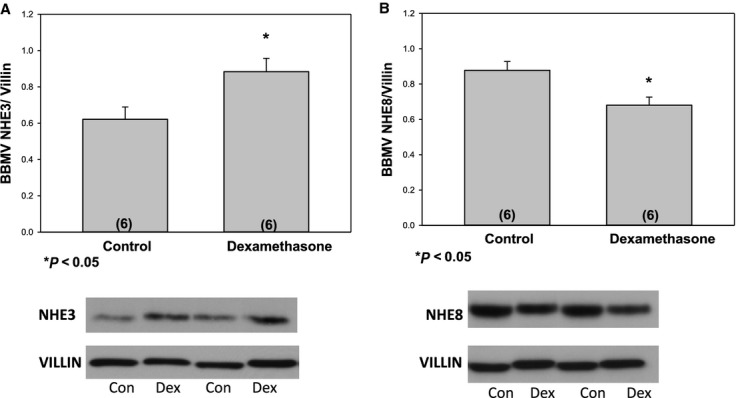
Effect of dexamethasone on neonatal renal NHE3 and NHE8 brush border membrane protein abundance. Brush border membrane vesicles were harvested from 10-day-old kidneys after mice received either vehicle or dexamethasone subcutaneously for 3 days. Dexamethasone resulted in an increase in brush border membrane NHE3 (A) and decrease NHE8 abundance (B) in brush border membranes.

The Na^+^/H^+^ isoform switch from NHE8 to NHE3 occurs around the time of weaning and NHE3 and NHE8 mRNA abundance are fairly constant for the first 2 weeks of life (Twombley et al. 2010). Administration of daily dexamethasone for 3 days starting on day 7 of life resulted in an increase in NHE3 mRNA abundance and decrease in NHE8 mRNA abundance compared with vehicle-treated controls (Fig. 1). We next examined if the increase in NHE3 mRNA was translated to an increase in NHE3 protein abundance and if the decrease in NHE8 mRNA abundance resulted in a decrease in NHE8 total protein abundance. As shown in Figure 2, there was an increase in total protein NHE3 abundance in the dexamethasone-treated mice and a concomitant decrease in NHE8 total protein abundance. Finally, we examined if dexamethasone affected brush border membrane protein abundance in neonates. As shown in Figure 3, dexamethasone administration increased NHE3 and decreased NHE8 brush border membrane protein abundance compared with vehicle-treated controls. Thus, dexamethasone resulted in an increase in NHE3 and decrease in NHE8 mRNA and protein abundance before the normal maturational change consistent with glucocorticoids regulating both Na^+^/H^+^ exchangers but in the opposite direction.

**Figure 4:**
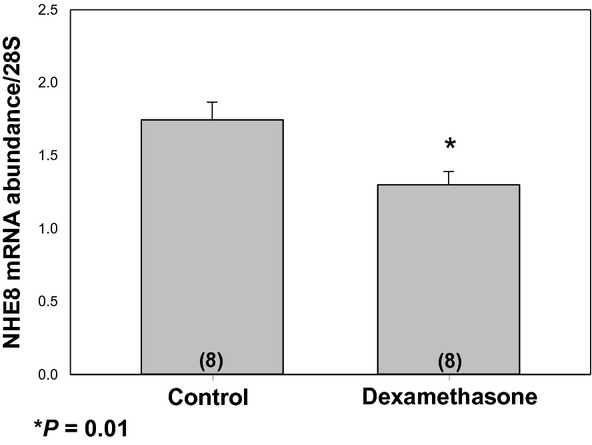
Effect of dexamethasone on NHE8 mRNA abundance in NRK cells. NRK cells were grown to confluence and then rendered quiescent. Vehicle or 10^−6^ mol/L dexamethasone was added to the media and the cells were studied 24 h later. NHE8 mRNA abundance was measured using real-time PCR. The abundance of NHE8 mRNA was less in the dexamethasone-treated cells.

**Figure 5:**
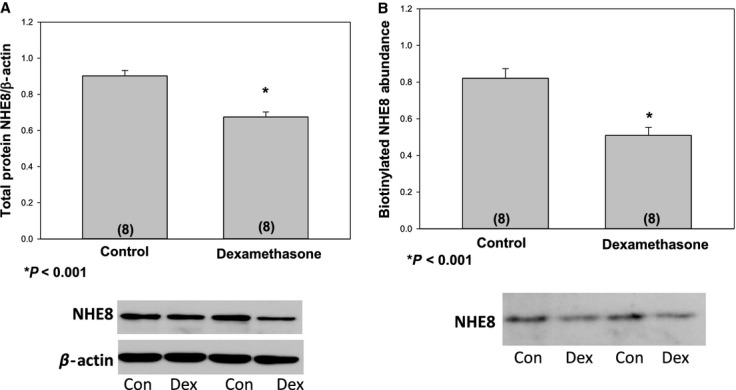
Effect of dexamethasone on NHE8 surface protein abundance in NRK cells. Confluent NRK cells were processed for total protein abundance (A) or were surface biotinylated and surface proteins harvested using streptavidin-agarose beads (B). NHE8 total protein and surface protein abundance was less in dexamethasone-treated cells than control.

We had previously shown that glucocorticoids, in addition to their hemodynamic action, increased NHE by a direct epithelial action in OKP cells which express NHE3 (Baum et al. 1993; Cano et al. 1999). To test if there was a direct epithelial action of dexamethasone on NHE8 abundance and activity, we studied NRK cells which express NHE8 but not NHE3 (Zhang et al. 2007). As we demonstrated in vivo, dexamethasone decreased NHE8 mRNA abundance as shown in Figure 4. Dexamethasone also decreased NHE8 total protein and surface protein expression which is shown in Figure 5.

**Figure 6:**
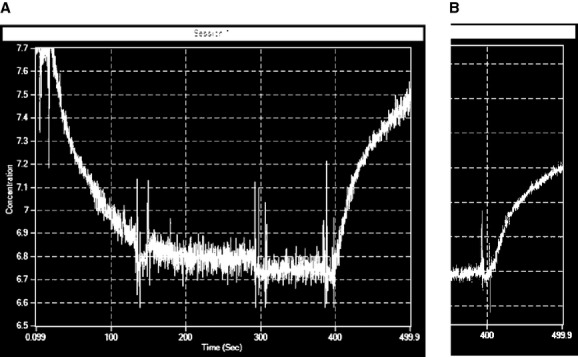
Typical tracing of Na^+^/H^+^ exchanger activity in NRK cells. NRK cells were grown on glass cover slips and treated with vehicle (A) or dexamethasone for 24 h (B). Na^+^/H^+^ exchange activity was measured as the rate of pHi recovery after an acid load using the K nigericin technique. The rate of pH recovery upon addition of sodium at 400 sec is due to Na^+^/H^+^ exchange.

**Figure 7:**
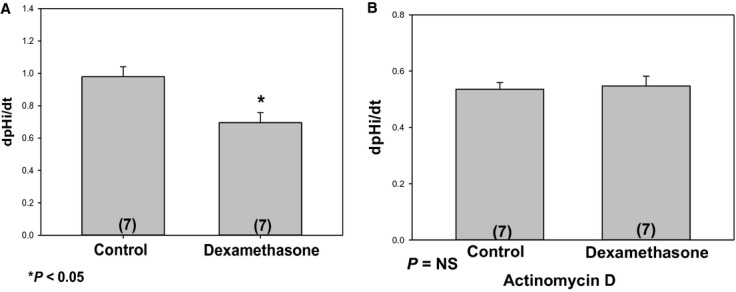
Effect of dexamethasone on Na^+^/H^+^ exchanger activity in NRK cells. Dexamethasone-treated cells had a slower rate of Na^+^/H^+^ exchange activity as shown in (A). However, when Na^+^/H^+^ exchange was assessed in the presence of actinomycin D, there was no difference between dexamethasone- and vehicle-treated cells (B).

To determine if glucocorticoids regulate Na^+^/H^+^ exchanger activity, we examined the effect of pH recovery after an acid load. A typical tracing is shown in Figure 6. We found no effect of dexamethasone at 6 and 12 h (not shown) but a significant decrease in NHE activity in dexamethasone-treated cells at 24 h (Fig. 7). The inhibitory effect of dexamethasone on NHE activity was blocked by actinomycin D (Fig. 7).

## Discussion

This study examined if glucocorticoids had an effect on the neonatal kidney that may explain the maturational decrease in NHE8 expression with a concordant increase in NHE3 expression. We found that administration of dexamethasone before the developmental isoform change at the time of weaning resulted in a decrease in brush border membrane NHE8 and increase in NHE3 expression. As glucocorticoids may alter hemodynamics and other factors that may decrease NHE8 expression, we also examined if there was a direct epithelial effect of dexamethasone using NRK cells that express NHE8, but not NHE3 (Zhang et al. 2007). We demonstrate that dexamethasone has a direct epithelial action to decrease NHE8 mRNA abundance and surface protein expression as well as Na^+/^H^+^ exchanger activity.

Previous studies in adult animals have demonstrated that glucocorticoids have a number of actions that affect acid–base balance. Glucocorticoids increase net acid production and net acid and ammonia excretion (Welbourne et al. 1976; Hulter et al. 1980). Despite the increase in acid production, glucocorticoids increase serum bicarbonate levels (Welbourne et al. 1976; Hulter et al. 1980), which is due in large part to the effect of glucocorticoids to increase Na^+^/H^+^ exchanger activity (Freiberg et al. 1982; Kinsella et al. 1984, 1985). Administration of dexamethasone to pregnant rabbits has been shown to increase neonatal NHE activity and rate of proximal tubule bicarbonate absorption (Baum and Quigley 1991). Glucocorticoids can have hemodynamic effects which could secondarily increase Na^+^/H^+^ exchanger activity, but they also have a direct epithelial action to increase proximal tubule acidification (Baum and Quigley 1993a; Baum et al. 1993).

Previous studies have examined the effect of glucocorticoids on NHE3, the predominant Na^+^/H^+^ exchanger on the brush border membrane in adults. Administration of glucocorticoids to neonatal rabbits increases renal cortical NHE3 mRNA and protein abundance (Baum et al. 1995), whereas glucocorticoid deficiency in the neonatal rat attenuates the developmental increase in Na^+^/H^+^ exchanger activity but has no effect on NHE3 mRNA abundance (Gupta et al. 2001). While the chronic effect of glucocorticoids on NHE3 is likely predominantly due to an increase in transcription, the acute effect of glucocorticoids occurs without a change in NHE3 mRNA abundance (Bobulescu et al. 2005). The acute activation of NHE3 by glucocorticoids requires phosphorylation of the NHE3 by SGK1 which occurs before an increase in mRNA abundance (Yun et al. 2002; Wang et al. 2005). The acute increase in Na^+^/H^+^ exchanger activity is paralleled by an increase in surface expression in OKP cells and is due to an increase in NHE3 exocytosis (Bobulescu et al. 2005).

The maturational increase in bicarbonate absorption and Na^+^/H^+^ exchanger activity paralleled the developmental increase in plasma thyroid hormone and glucocorticoid levels (Wysocki and Segal 1972; Henning 1978; Walker et al. 1980). This increase in thyroid hormone and glucocorticoid levels has been examined as a potential factor responsible for the increase in proximal tubule acidification during postnatal development. Thyroid hormone has been shown to positively regulate renal Na^+^/H^+^ exchanger activity in BBMV (Kinsella and Sacktor 1985; Kinsella et al. 1986), which is due to a direct epithelial effect to increase Na^+^/H^+^ exchanger activity via an increase in transcription of NHE3 (Yonemura et al. 1990; Cano et al. 1999). The maturational increase in NHE3 mRNA and protein abundance and Na^+^/H^+^ exchanger activity can be accelerated by administration of thyroid hormone to neonates (Baum et al. 1998). We have also examined if thyroid hormone was a potential factor causing the reduction in NHE8 (Gattineni et al. 2008). Administration of thyroid hormone to neonatal rats resulted in a decrease in NHE8 protein but not mRNA abundance. Na^+^/H^+^ exchanger activity and NHE8 surface expression in NRK cells were inhibited by thyroid hormone demonstrating a direct epithelial effect of thyroid hormone on a proximal tubule cell line (Gattineni et al. 2008).

The present study examined the effect of glucocorticoids on NHE8 to determine if the maturational increase in glucocorticoid levels, which occurs at the time of weaning, could be a factor in the decrease in NHE8. However, other factors involved in glucocorticoid action may be developmentally regulated as well. There is evidence for developmental regulation of the glucocorticoid receptor in the kidney. Glucocorticoid receptor mRNA expression is higher in the neonatal than the adult rat kidney peaking at 14 days of life (Kalinyak et al. 1989). Renal glucocorticoid binding is detectable in the fetal mouse kidney and increases after birth reaching adult levels by 2 weeks of age (Ellis et al. 1986).

It is theoretically possible that the high dose of dexamethasone used in this study could act via the mineralocorticoid receptor. Whether there is a mineralocorticoid receptor in the proximal tubule has been examined previously and the results are conflicting. There are some studies consistent with very low mineralocorticoid receptor mRNA expression in the proximal tubule compared to the distal nephron (Todd-Turla et al. 1993), while other studies did not find evidence for the mineralocorticoid receptor in the proximal tubule or found that the effect of aldosterone on proximal tubule transport was due to binding to the glucocorticoid receptor (Roland et al. 1995; Pergher et al. 2009; Pao et al. 2010; Braga-Sobrinho et al. 2012). Thus, it is unlikely that the effect of dexamethasone in this study is via the mineralocorticoid receptor.

Glucocorticoids have been shown to affect the transport of many solutes that occur during postnatal development. Administration of glucocorticoids increases volume absorption, glucose transport, renal acidification, and Na^+^/K^+^ ATPase activity (Aperia et al. 1981; Beck et al. 1988; Baum and Quigley 1991, 1993a; Baum et al. 1994; Gupta et al. 2001). Not all solute transport increases during postnatal maturation. Phosphate transport decreases during postnatal development and glucocorticoids may play a role to cause this developmental change (Arar et al. 1994; Prabhu et al. 1997).

We have previously examined the ontogeny of NHE3 and NHE8 mRNA and protein abundance. In both the rat and the mouse there was an increase in both NHE3 mRNA and brush border membrane protein abundance (Becker et al. 2007; Twombley et al. 2010). The five- to 10-fold decrease in NHE8 brush border membrane protein expression in the rat and mouse was not reflected by a comparable change in mRNA abundance (Becker et al. 2007; Twombley et al. 2010). In the rat we found no developmental change in mRNA abundance while in the mouse there was a transient twofold increase in NHE8 mRNA abundance at 24 days of age but the NHE8 mRNA abundance was comparable in 1-, 7-, 14-day-old neonates as that in the adult. Thus, it appeared that most of the regulation of NHE8 was posttranscriptional. However, this study shows that dexamethasone decreases mRNA NHE8 abundance. In addition, the effect of dexamethasone on Na^+^/H^+^ exchanger activity was inhibited by actinomycin D in NRK cells. It is possible that glucocorticoids are counterbalancing another factor causing a maturational increase in NHE8 mRNA abundance resulting in no net effect or the transient increase at 24 days. The mechanism for how glucocorticoids decrease NHE8 transcription has been examined in intestine and in Caco-2 cells (Xu et al. 2010). Studies using rats found that glucocorticoids decrease both NHE8 mRNA and protein abundance in ileum and jejunum. In Caco-2 cells transfected with the NHE8 promotor, transcription activity was inhibited by glucocorticoids in vitro by enhanced binding of *Pax5* to the NHE8 promotor.

In the intestine and proximal tubule NHE8 is predominantly expressed on the brush border membrane (Goyal et al. 2003; Xu et al. 2005). A recent study examining the effect of NHE8 on adult renal acidification showed that NHE8^−/−^ mice had comparable apical Na^+^/H^+^ exchanger activity as wild type mice consistent with NHE3 playing the predominant role in luminal proton secretion in the adult proximal tubule (Baum et al. 2012). However, there was an increase in NHE8 brush border membrane protein abundance in NHE3^−/−^ mice compared with wild type mice and proximal tubule Na^+^/H^+^ exchanger activity was greater in NHE3^−/−^ mice than in NHE3^−/−^/NHE8^−/−^ mice consistent with NHE8 playing a compensatory role in luminal acidification in the absence of NHE3. The relative roles of NHE3 and NHE8 in neonatal proximal tubule acidification are not clear at present. In this study, we examined NHE3 and NHE8 protein and mRNA expression in kidneys from mice. This does not compare the functional activity of these two transporters nor explain why there is an isoform change during postnatal development. In summary, this study shows that glucocorticoids regulate NHE8 in vivo and in vitro and may be a factor in the renal isoform change during postnatal development.
